# Publisher’s Note: Continued Publication of *Pediatric Reports* by MDPI

**DOI:** 10.3390/pediatric12030016

**Published:** 2020-10-16

**Authors:** Franck Vazquez, Unai Vicario, Shu-Kun Lin

**Affiliations:** 1 MDPI, St. Alban-Anlage 66, CH-4052 Basel, Switzerland; lin@mdpi.com; 2 MDPI, Avenida Madrid, 95, 08028 Barcelona, Spain; vicario@mdpi.com

*Pediatric Reports* was launched in 2009 and it has been published over the past eleven years by PAGEPress Publications [1]. Professor Maurizio Aricò has served as its Editor-in-Chief since its inception [2] and is currently still active in this role. 

We are delighted to take over the publication of *Pediatric Reports* from PAGEPress and perpetuate the legacy of this journal, and ensure that we serve well the pediatrician community. *Pediatric Reports* complements very well the MDPI portfolio of medical journals [3], especially *Children* [4] and the *International Journal of Neonatal Screening* [5].

MDPI will publish the fourth quarterly issue of 2020 and will thereafter publish four quarterly issues as of 2021. 

Enjoy publishing your work in *Pediatric Reports* [6]!
